# Cholesterol and docosahexaenoic acid likely protect against alcohol-induced constriction of cerebral arteries via a common pathway

**DOI:** 10.3389/adar.2026.16302

**Published:** 2026-07-17

**Authors:** Shiwani Thapa, Steven Mysiewicz, Alex M. Dopico, Anna N. Bukiya

**Affiliations:** Department of Pharmacology, Addiction Science, and Toxicology College of Medicine, The University of Tennessee Health Science Center, Memphis, TN, United States

**Keywords:** binge drinking, fish oil, hypercholesterolemia, hyperlipidemia, MaxiK channel

## Abstract

Binge alcohol drinking is significantly associated with an increased risk of cerebrovascular dysfunction. Within cerebral artery smooth muscle, alcohol inhibits calcium- and voltage-gated potassium (BK) channels of large-conductance, leading to cerebrovascular constriction. Administration of high-cholesterol diet or docosahexaenoic acid (DHA) supplementation independently protects against alcohol-induced constriction of cerebral arteries. However, whether these interventions act additively or through a shared mechanism(s) remains unclear. Here, we examined the combined effects of cholesterol and DHA dietary supplementation *in vivo* and their combined administration *ex vivo* on alcohol-induced changes in rat cerebral artery diameter. DHA dietary supplementation *in vivo* and application to rat cerebral arteries *ex vivo* did not further modify protective action of high-cholesterol diet and cholesterol-enrichment, respectively. Lack of additivity may point to a shared mechanism(s) that drives cholesterol- and DHA-driven protection against alcohol constriction of cerebral arteries. Considering a prior report of BK channel α-subunit (slo1) residue tyrosine 450 mediating cholesterol protection against alcohol cerebrovascular action, we probed slo1Y450 role in DHA protection against alcohol effect on cerebral artery diameter. DHA protection against alcohol-induced constriction vanished in cerebral arteries harvested from male slo1Y450F knock-in mice. Yet DHA protection against alcohol was still present in arteries harvested from female slo1Y450F knock-in mice. Combined with previous observations, our current findings identify slo1Y450 as a potentially shared target involved in a sexually dimorphic pathway between alcohol effect on cerebral artery diameter and its sensitivity to cholesterol- and DHA-driven interventions.

## Introduction

Binge drinking is the most common pattern of excessive alcohol consumption in the United States [1]. Binge drinking renders blood alcohol levels exceeding 80 mg/dL (or 17 mM) and is associated with widespread tissue and organ damage [2]. In the brain, high concentrations of alcohol induce constriction of cerebral arteries in a wide variety of preclinical models and humans [3, 4]. Alcohol-induced constriction of cerebral arteries has long been proposed as a contributing factor to the increased risk of stroke following excessive alcohol consumption [3, 5]. Thus, identifying viable strategies to mitigate alcohol-induced constriction of cerebral arteries is much needed.

Alcohol-induced constriction of cerebral arteries is enabled by alcohol inhibition of calcium- and voltage-gated potassium channels of large conductance (BK channels) in cerebrovascular myocytes [4, 6]. In smooth muscle (such as cerebrovascular wall), BK channels are activated by calcium mobilization during membrane depolarization [7, 8]. Activation of BK channels generates outward potassium currents that repolarize membrane back to resting potential, oppose myocyte contraction and favor vasodilation [9]. Thus, BK channel inhibition by alcohol triggers cerebrovascular constriction [4].

Using rodent models, our group previously reported surprising protection of high-cholesterol diet against cerebrovascular constriction by alcohol [10]. This protective effect of cholesterol is mediated at the level of cerebrovascular smooth muscle [10]. Moreover, it requires the cellular environment of cerebrovascular myocytes kept intact [11] and is ablated by Y450F mutation in the BK channel-forming alpha subunit (slo1) [12]. Recently, we also identified the polyunsaturated fatty acid docosahexaenoic acid (DHA) as dietary protectant against alcohol-induced constriction of cerebral arteries in males [13]. DHA-mediated protection against alcohol in cerebral arteries also requires the intact cellular environment of cerebrovascular myocyte and the presence of BK channel-forming alpha subunits [13]. However, important gaps remain in our understanding of how dietary factors interact with alcohol to influence cerebrovascular function; it is unknown whether DHA supplementation in the context of a high-cholesterol diet confers additive or synergistic protection against alcohol-induced constriction of cerebral artery diameter. Moreover, the distinct amino acid(s) within the BK channel alpha subunit that enable(s) DHA-mediated protection against alcohol-induced vasoconstriction has yet to be identified. Addressing these questions will advance mechanistic understanding of alcohol–diet interactions at the cerebrovascular level and may identify novel molecular targets for strategies aimed to reduce alcohol-related vascular risk.

## Materials and methods

### Animals and ethical considerations

All animal procedures reported in this manuscript were performed upon approval by the Institutional Animal Care and Use Committee. The University of Tennessee Health Science Center is accredited by the Association for Assessment and Accreditation of Laboratory Animal Care.

### Dietary interventions

Sprague-Dawley rats (50 g) of both sexes were purchased from Envigo and acclimated for 3 days upon arrival to the University of Tennessee Health Science Center. Then, animals were placed on a high-cholesterol diet (2% cholesterol) in standard rodent food (TD.07684, Harland-Teklad) *ad libitum*. Rats were used for experimentation after 18 weeks on high-cholesterol diet. By this point, rat blood lipid panel is known to exhibit a significant elevation of total cholesterol due to increase in low-density lipoprotein count [10]. Cholesterol amount per mg of protein within cerebral arteries also increases significantly [10]. While being placed on high-cholesterol diet, rats were randomized into DHA-receiving versus control groups. Animals in the DHA supplementation group received daily oral administration of DHA into the oral cavity at 14 mg/kg of weight using pipette dispenser as described [13]. Placebo group was receiving an equivalent volume of water dispensed via pipette. Daily DHA supplementation results in sexually dimorphic (males only) increases in DHA level per mg of protein within cerebral arteries while females have already higher DHA levels prior to receiving DHA regimen [13].

### Cranial window *in vivo*

Cranial window experiments were performed as described by our group [14]. Briefly, male and female rats were initially anesthetized with ketamine/xylazine mixture (91/9 mg/kg of weight, IP injection). Every 15 min, lack of sensation was verified with toe pinch and maintenance ketamine dose was injected (50 mg/kg of weight). Females in estrus were excluded from experimentation until they reach next stage of the estrous cycle. A catheter was inserted in the carotid artery using standard veterinary technique to ensure that the infused alcohol was directed toward cerebral circulation. A cranial window was then made on the side where the catheter had been inserted. The area above the zygomatic arch between the ear and the eye was cleared of hair using Nair hair removal cream. Skin and underlying tissue were dissected out. Then, the bone was removed using a Dremel 4000 instrument. Middle cerebral artery pial branches were monitored using a Leica MC170 HD microscope with a mounted camera (M125 C) connected to a computer monitor. Alcohol was diluted to reach 50 mM in 0.9% NaCl and administered via bolus injection into catheter at 1 mL/250 g of weight at a rate 1 mL/min. Cranial window images before and after alcohol infusion administration were acquired every 60 s. At the end of the experiment rats were euthanized via thoracotomy.

### Cerebral artery diameter monitoring *ex vivo*

Rats and mice were used as tissue donors. Sprague-Dawley rats (250–300 g) were purchased from Envigo and acclimated for at least 3 days upon arrival at the University of Tennessee Health Science Center. Slo1Y450F knock-in (KI) breeders were initially acquired from the laboratory of Dr. Gregg Homanics (University of Pittsburgh). Mouse colony was then established at the University of Tennessee Health Science Center where mice were bred to render wild-types (C57BL/6J) and slo1Y450F KI on C57BL/6J background from the same parental pair. For cerebral artery diameter monitoring *ex vivo*, 8–12 weeks-old mice were used.

On the day of the experiment, vaginal swabs were performed to establish the stage of estrous cycle in females. Similar to cranial windows *in vivo*, females in estrus were excluded from experimentation *ex vivo* until they reached the next stage of the estrous cycle. For cerebral artery harvesting, animals were deeply anesthetized with isoflurane using drop in a jar approach. Upon loss of toe pinch sensitivity, rats and mice were decapitated using guillotine and sharp scissors, respectively. Brains were immediately placed into an ice-cold physiological saline solution (PSS) of the following composition (mM): 119 NaCl, 4.7 KCl, 1.2 KH_2_PO_4_, 1.6 CaCl_2_, 1.2 MgSO_4_, 0.023 EDTA, 11 glucose, 24 NaHCO_3_. Middle cerebral arteries were dissected out under microscope (Nikon SMZ645) and de-endothelialized by passing an air bubble into the artery lumen for 90 s. First, one end of the artery was cannulated on a hollow glass cannula within perfusion chamber, this cannula was connected to plastic tubing through which air bubble was pushed to remove endothelium. After this, physiological saline was perfused through the artery lumen in a gentle manner to flush cellular debris. This approach has been validated to remove functional endothelium by the lack of arterial dilation in response to the endothelium-dependent vasodilator carbachol as opposed to endothelium-independent vasodilator sodium nitroprusside [4]. Immediately following de-endothelialization, artery’s second end was cannulated on the other cannula within perfusion chamber (Living Systems Instrumentation). The chamber was continuously perfused at a rate of 1.65 mL/min with PSS that was being equilibrated at pH 7.4 with a 21/5/74% mix of O_2_/CO_2_/N_2_ gases and maintained at 35 °C–37 °C. Artery diameter was monitored via camera attached to an inverted microscope (Nikon Eclipse TS100) with a 10X objective. The external wall diameter was continuously monitored and plotted on a computer screen over time using the automatic edge-detection software (IonWizard by IonOptics). When required for pressurization, intralumenal pressure was changed using different levels of elevation of attached reservoir filled with PSS. Changes in pressure were monitored using a pressure transducer (Living Systems Instruments). Arteries were first incubated at an intravascular pressure of 10 mm Hg for 10 min. Then, intravascular pressure was increased to 60 mmHg and held steady throughout the experiment to trigger development and ensure maintenance of the myogenic tone. The latter was considered present if arteries responded to intralumenal pressure with constriction [15]. Continuous dilation of the artery in response to intralumenal pressure (60 mmHg) was used as a criterion to remove such recording from analysis. Drugs from stocks were prepared in PSS to reach their desired concentration. Drug-containing PSS was continuously pumped into artery perfusion chamber.

### Chemicals

DHA ethyl ester for feeding *in vivo* was purchased from Sigma Aldrich. Single-use aliquots of DHA for experiments *ex vivo* were purchased from Cayman. Cholesterol, methyl-β-cyclodextrin (MβCD), ethanol (ethyl alcohol, 190 proof) and all other chemicals were purchased from Sigma-Aldrich.

On the day of the experiment, ethanol was diluted to 50 mM. For cranial windows *in vivo* and pressurized arteries *ex vivo*, ethanol was diluted in 0.9% NaCl and PSS, respectively. For enrichment of cerebral arteries with cholesterol *ex vivo*, we followed previously described method [12, 16, 17]. Briefly, cholesterol powder was added to MβCD in PSS to render 0.625:5 mM cholesterol:MβCD (1:8) molar ratio. Resulting suspension was incubated on shaking water bath at 37 °C overnight and filtered the next morning prior to use. Pressurized cerebral arteries were perfused extraluminally with cholesterol:MβCD complex for 15 min to render cholesterol elevation within artery wall [12, 16]. For DHA perfusion *ex vivo*, stock solution of DHA was first prepared in dimethyl sulfoxide (DMSO), and then further diluted in PSS to the desired concentration. Final content of DMSO in artery bathing solution did not exceed 0.04%.

### Statistical analysis and data plotting

Analysis of cranial window images obtained *in vivo* was performed using ImageJ software by the investigator who was blind to experimental group identity. After all diameter measurements were documented, animal group identity was revealed and data from different rats were grouped accordingly. Diameter traces obtained from pressurized cerebral arteries *ex vivo* were analyzed using IonWizard 4.4 software (IonOptix). Pressurized artery basal diameter (i.e., diameter before drug application) was determined by averaging diameter values during 3 min of continuous recording immediately before drug application. Artery diameter in presence of a specific drug was determined immediately before the perfusion was switched to a washout. Alcohol-induced constriction was measured as percent change of artery diameter by alcohol from basal diameter (pre-alcohol) level.

Statistical analysis was performed using free online tool Statskingdom. With number of observations in experimental groups <10, the mode of data distribution could not be established with certainty. Thus, non-parametric Mann-Whitney test (2-tailed) was used to establish statistical significance. For datasets with n ≥ 10, testing of the mode of distribution (Gaussian versus non-Gaussian) was performed using the free online tool Statskingdom. If distribution was Gaussian, unpaired t-test (2-tailed) was used to determine significance of differences between the groups. If data distribution was not Gaussian, statistical significance was probed using non-parametric Mann-Whitney test (2-tailed). Data from mouse arteries was analyzed by two-way analysis of variance (ANOVA) with genotype (wild-type versus slo1Y450F KI) and treatment (DHA versus vehicle control) as independent factors. In all cases, statistical significance was set at p < 0.05.

Final plotting of data was conducted using Origin 2023 software (OriginLab). Data are expressed as mean ± standard error.

## Results

### *In vivo* dietary DHA supplementation combined with high-cholesterol diet does not modify the response of cerebral arteriole diameter to alcohol when compared to high-cholesterol diet without DHA

**FIGURE 1:**
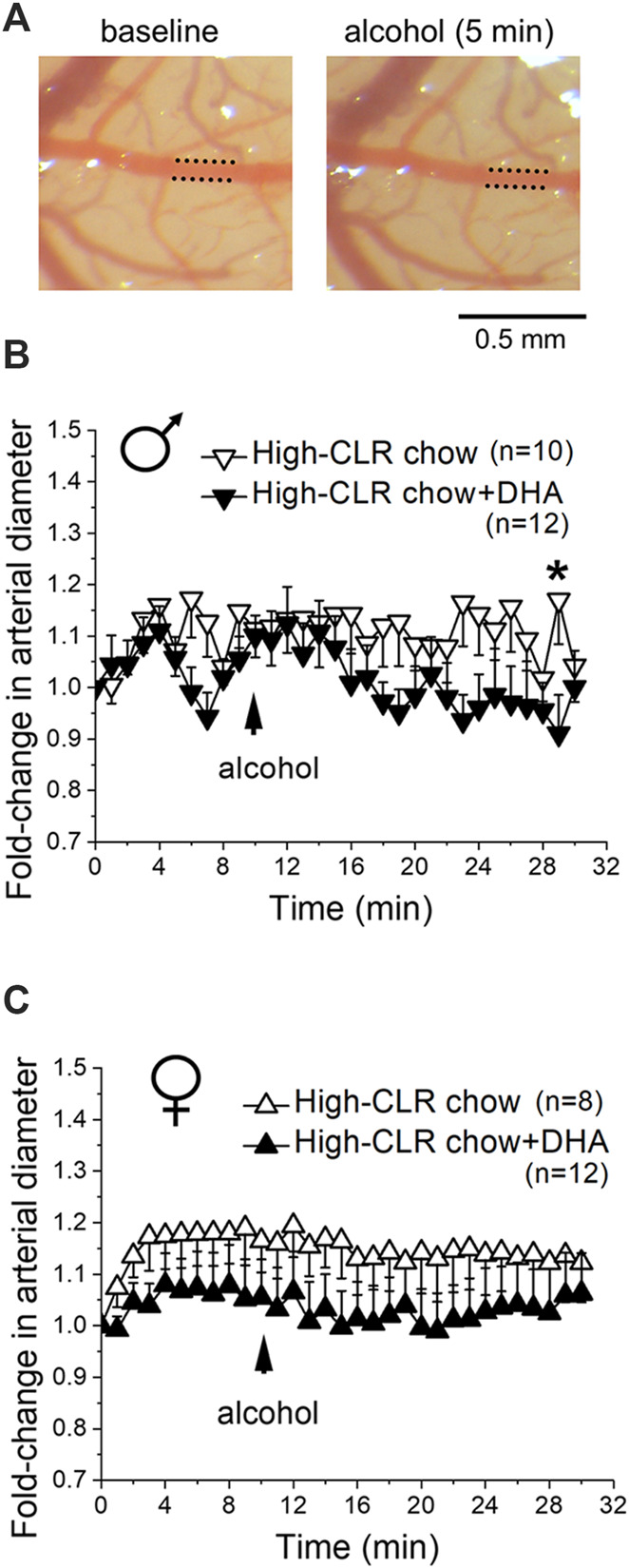
Dietary DHA supplementation combined with high-cholesterol diet does not modify response of cerebral arteriole diameter to alcohol administration *in vivo* when compared to high-cholesterol diet alone. **(A)** Example of original photographs obtained from female rat receiving high-cholesterol diet combined with daily DHA oral gavages. Photographs depict arterioles at time-point zero (baseline, panel at the left) versus arterioles after 5 min following alcohol infusion. Dotted lines depict arteriole external diameter under observation and subsequent analysis. **(B)** Averaged data showing fold-change in arteriole diameter from baseline (time-point zero) over time in male rats receiving high-cholesterol diet with daily DHA oral supplementation versus control. *p = 0.03375 by 2-tail unpaired t-test. **(C)** Averaged data showing fold-change in arteriole diameter from baseline (time-point zero) over time in female rats receiving high-cholesterol diet with daily DHA oral supplementation versus control.

We have previously established that either high-cholesterol diet or DHA oral supplementation protects against alcohol-induced constriction of cerebral arteries [10, 13]. To determine whether combining high-cholesterol diet with daily DHA oral supplementation offers additional benefit against alcohol effect on cerebral artery diameter, we performed cranial windows on rats *in vivo*. Prior to cranial window surgery, rats were maintained on a high-cholesterol diet (2% cholesterol) for 18 weeks *ad libitum*. For the duration of high-cholesterol diet, experimental groups of males versus females also received daily gavages with DHA. In control groups, daily supplementation via gavage consisted of distilled water that was isovolumic to gavages with DHA as quantified per animal’s weight. After cranial window installation, arteriole diameter was documented every minute, with changes in arteriole diameter over time being presented as a fold-change from initial diameter, that is, the diameter observed during 1st minute of observation (Figure 1). Baseline variations in arteriole diameter were established over the course of 10 min. Then, alcohol was probed at 50 mM injected into cerebral circulation via catheter in the carotid artery. As expected, high-cholesterol diet protected against alcohol-induced constriction. In males, fold-change in arteriole diameter from the initial timepoint only reached 1.13 ± 0.09 nine minutes after alcohol was infused (Figure 1B). In females, this value was 1.12 ± 0.08 (Figure 1C). Both male and female values of fold-change in diameter by alcohol were not statistically different from baseline variations.

In experimental group of males that were receiving daily DHA oral gavages during the course of high-cholesterol diet, fold-change in arteriole diameter from the initial timepoint averaged 0.95 ± 0.04 nine minutes following alcohol infusion, and it was not statistically different from control group (high-cholesterol diet with daily gavages consisting of distilled water) (Figure 1B). Of note, fold-changes in artery diameter from the initial timepoint were statistically lower in DHA group at a single time point following alcohol infusion (20 min, Figure 1B). In experimental group of females that were receiving daily DHA oral gavages during the course of high-cholesterol diet, fold-change in arteriole diameter from the initial timepoint averaged 1.04 ± 0.07 nine minutes following alcohol infusion, and it was not statistically different from control group (high-cholesterol diet supplemented with daily gavages consisting of distilled water) (Figure 1C). We did not detect statistically significant differences in fold-change arteriole diameter from the initial timepoint between control and DHA groups of females, albeit averaged values in DHA group were consistently lower than in control (Figure 1C).

In neither males nor females, DHA oral supplementation during a high-cholesterol diet exerted a clear effect on alcohol-induced changes in arteriole diameter compared to control group. These findings suggest that cholesterol- and DHA-driven protection of cerebral artery against alcohol-induced constriction may converge on a shared mechanistic pathway (ceiling effect).

### *Ex vivo* DHA combined with cholesterol-enrichment does not modify response of cerebral artery diameter to alcohol when compared to cholesterol-enrichment without DHA

We have previously established that either cholesterol-enrichment of arteries *ex vivo* or their bathing in DHA-containing solution protects against alcohol-induced constriction of cerebral arteries [10, 13]. To determine whether combining cholesterol-enrichment *ex vivo* with application of DHA-containing solution offers additional benefit against alcohol effect on cerebral artery diameter, we probed pressurized middle cerebral arteries *ex vivo*. Middle cerebral arteries from rats were dissected out and pressurized to 60 mmHg to develop myogenic tone and allow assessment of pharmacological responses under physiologically relevant conditions [4, 18]. After arteries developed myogenic tone, they were enriched with cholesterol by incubation in a MβCD-cholesterol complex as described [16]. This procedure increases artery cholesterol content 1.5–2.0-fold [12, 16], and was previously shown to protect middle cerebral arteries of rat against alcohol-induced constriction [10]. Moreover, cholesterol level within artery wall remains elevated after MβCD-cholesterol bathing is replaced with regular physiological saline solution. Following removal of MβCD-cholesterol complex from the bath, cholesterol-enriched arteries were bathed in 3 μM DHA, a concentration previously shown to protect against alcohol-induced constriction of *ex vivo* pressurized middle cerebral arteries from rat [13]. Artery diameter prior to DHA probing in males and females averaged 174 ± 5 μm (n = 33) and 217 ± 8 μm (n = 26), respectively. These diameters did not differ significantly from baseline diameters before DMSO application (see below) which averaged 171 ± 5 μm in males (n = 45) and 198 ± 8 μm in females (n = 32) (p = 0.71 and 0.1 by 2-tail t-test for DHA versus DHA groups from males and females, respectively). Alcohol was probed at physiologically and toxicologically relevant concentrations as follows: 5, 17.5, 50, 75, and 100 mM. In control group (cholesterol-enrichment without DHA), alcohol was probed in a manner that was time-matched to experimental group. Moreover, vehicle control to bathing with DHA (0.04% DMSO) was probed with 17.5, 50, and 100 mM alcohol. Vehicle perfusion (0.04% DMSO) did not alter baseline arterial diameter or alcohol responses.

**FIGURE 2:**
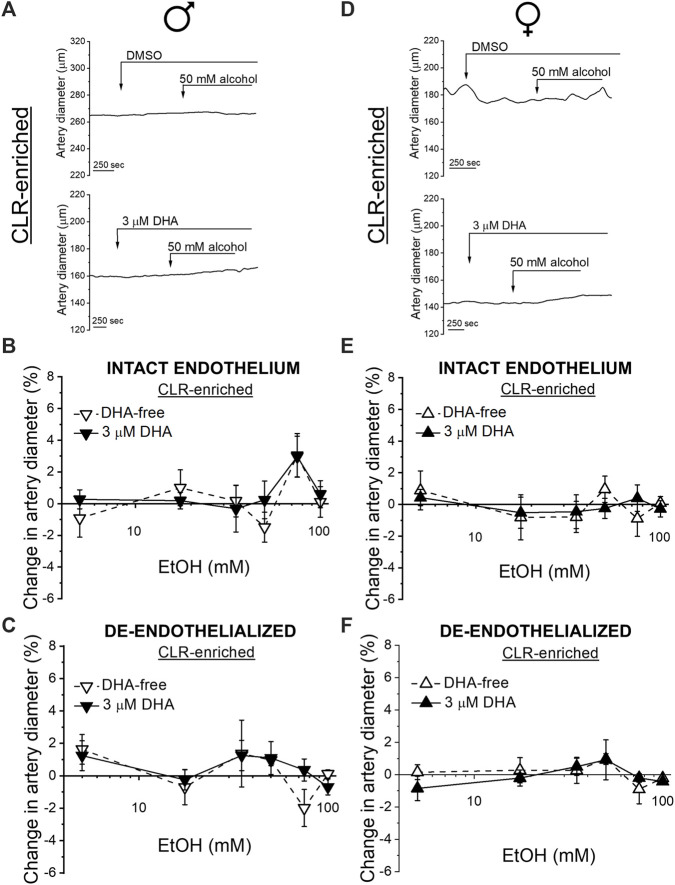
DHA combined with cholesterol-enrichment does not modify response of cerebral artery diameter to alcohol *ex vivo* when compared to cholesterol-enrichment without DHA. **(A)** Example of artery diameter traces obtained from male rat middle cerebral arteries. **(B)** Averaged data from male rats showing percent change in diameter by alcohol from diameter immediately prior to alcohol application *ex vivo*. **(C)** Averaged data from male rats showing percent change in diameter by alcohol from diameter immediately prior to alcohol application *ex vivo* (deendothelialized arteries). **(D)** Example of artery diameter traces obtained from female rat middle cerebral arteries. **(E)** Averaged data from female rats showing percent change in diameter by alcohol from diameter immediately prior to alcohol application *ex vivo*. **(F)** Averaged data from female rats showing percent change in diameter by alcohol from diameter immediately prior to alcohol application *ex vivo* (deendothelialized arteries).

As expected, high-cholesterol diet precluded alcohol-induced constriction across the entire range of alcohol concentrations in both sexes (Figures 2A,B,D,E). Addition of DHA did not produce statistically significant changes in cerebral artery diameter beyond those observed with cholesterol-enrichment alone despite the fact that our recent observations using the same experimental setting in cholesterol-naïve (not subjected to any manipulation on naturally occurring cholesterol levels) arteries clearly pointed to a protective effect of DHA against alcohol-induced constriction in middle cerebral arteries from male Sprague-Dawley rats [13]. This lack of additive or synergistic effect further reinforces the idea that cholesterol- and DHA-driven protection against alcohol constriction may converge on a shared mechanism. Moreover, that lack of additivity in cholesterol and DHA action during *in vivo* and *ex vivo* testing suggests that common pathway of interaction with alcohol is independent of systemic circulation and complex neuronal integrity.

To assess the contribution of endothelial-derived vasoactive factors into this pathway, experiments were repeated following physical removal of the endothelium by intraluminal passing of air bubbles [4, 19]. Artery diameter prior to DHA probing in males averaged 179 ± 7 μm (n = 30), while in females it was 207 ± 10 μm (n = 30). Artery diameter prior to DMSO probing in males averaged 172 ± 6 μm (n = 42), while in females it was 206 ± 10 μm (n = 34). There were no statistically significant differences between these groups (p = 0.48 by 2-tail t-test for DHA versus DHA groups from males; p = 0.85 by 2-tail Mann-Whitney test for DHA versus DHA groups from females). Removal of the endothelium and probing of cholesterol, DHA, and alcohol in deendothelialized arteries (Figures 2C,F) rendered an outcome that was similar to data obtained from arteries with intact endothelium (Figures 2B,E): cholesterol enrichment precluded alcohol-induced constriction across the entire range of alcohol concentration in both sexes, and DHA application to cholesterol-enriched arteries did not evoke any changes to lack of artery response to alcohol (Figures 2C,F). These findings indicate that the potentially shared mechanism underlying cholesterol- and DHA-dependent protection against alcohol-induced cerebrovascular constriction does not require a functional endothelium.

### Role of Y450 within cerebrovascular BK channel-forming α subunits in enabling DHA protection against alcohol

Based on the hypothesis that cholesterol and DHA share a common mechanism underlying their protection against cerebral artery constriction by alcohol, we examined the role of tyrosine 450 (Y450) in the cerebrovascular BK channel-forming α subunit (slo1). Our previous work showed that DHA-driven protection against alcohol-induced constriction of cerebral arteries required the presence of BK channel-forming α subunits [13]. Notably, tyrosine 450 substitution to phenylalanine (Y450F) in this subunit disabled cholesterol-dependent protection against alcohol [12]. If cholesterol- and DHA-driven protections against alcohol shared a molecular pathway, then slo1Y450 would be a strong candidate to mediate DHA protection against alcohol constriction of cerebral arteries. If proven to mediate DHA protection against alcohol, slo1Y450 could then be postulated as a key structural mediator upon which cholesterol, DHA and alcohol actions on cerebral arteries converge.

**FIGURE 3:**
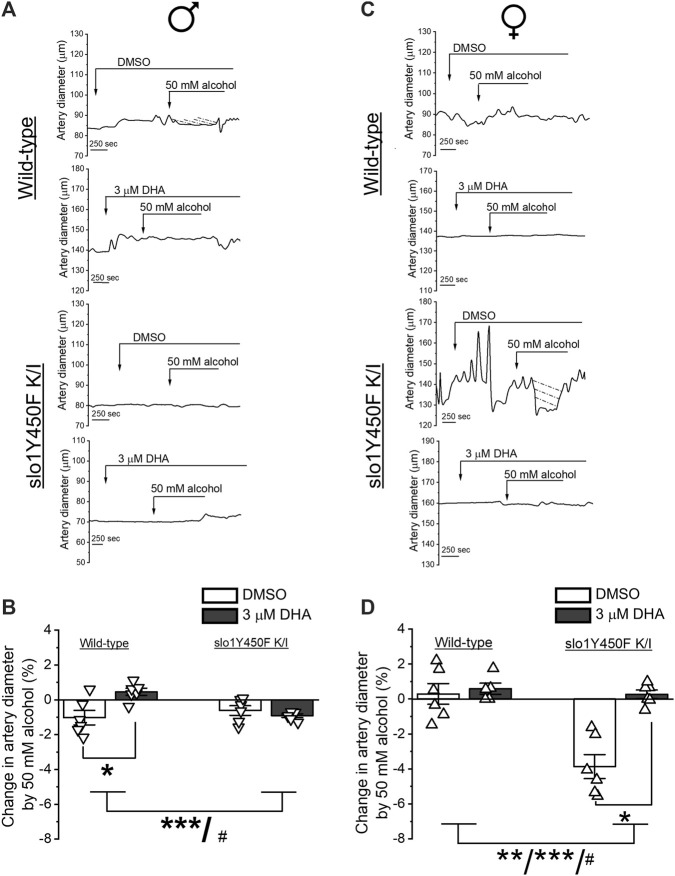
Role of Y450 within cerebrovascular BK channel-forming subunit (slo1) in DHA-driven protection against alcohol effect on cerebral artery diameter. **(A)** Original traces showing changes of middle cerebral artery dimeter by 50 mM alcohol *ex vivo (*deendothelialized arteries from males). **(B)** Scattered graph showing percent change in male middle cerebral artery external diameter by 50 mM alcohol in absence versus presence of DHA. Data is shown as mean ± SEM. *p < 0.05 by two-tailed Mann–Whitney test, ***p < 0.05 by two-way ANOVA (factor: treatment) and #p < 0.05 by two-way ANOVA (interaction between genotype and treatment). **(C)** Original traces showing changes in middle cerebral artery dimeter by 50 mM alcohol *ex vivo* (deendothelialized arteries from females). **(D)** Scattered graph showing percent change in female middle cerebral artery external diameter by 50 mM alcohol in absence versus presence of DHA. Data is shown as mean ± SEM. *p < 0.05 by two-tailed Mann–Whitney test, **p < 0.05 by two-way ANOVA (factor: genotype), ***p < 0.05 by two-way ANOVA (factor: DHA treatment) and #p < 0.05 by two-way ANOVA (interaction between genotype and treatment).

To test the role of Tyr450 in DHA-driven protection against alcohol, middle cerebral arteries isolated from C57BL/6J mice expressing wild-type BK channel (slo1) and global knock-in with slo1Y450F substitution on C57BL/6J background were pressurized *ex vivo* at 60 mmHg. Endothelium was removed by passing an air bubble through artery lumen immediately prior to pressurization. Following development of myogenic tone, there were no differences in average diameter between strain and sexes assigned to DHA versus DMSO probing. Namely, artery diameters in C57BL/6L males in DHA group averaged 123 ± 1 μm (n = 8) versus 125 ± 13 μm in DMSO group (n = 7) (p = 0.91 by 2-tail Mann-Whitney test). Artery diameters in slo1Y450F males in DHA group averaged 104 ± 11 μm (n = 7) versus 100 ± 17 μm in DMSO group (n = 6) (p = 0.73 by 2-tail Mann-Whitney test). Artery diameters in C57BL/6L females in DHA group averaged 132 ± 17 μm (n = 6) versus 107 ± 11 μm in DMSO group (n = 6) (p = 0.34 by 2-tail Mann-Whitney test). Artery diameters in slo1Y450F females in DHA group averaged 130 ± 18 μm (n = 6) versus 131 ± 2 μm in DMSO group (n = 6) (p = 0.48 by 2-tail Mann-Whitney test). Arteries were perfused with either 3 μM DHA or vehicle control (0.04% DMSO). Alcohol was probed at 50 mM, and alcohol-induced constriction was measured as percent change of artery diameter from pre-alcohol levels. In wild-type male mice, DHA protected against alcohol-induced constriction. Namely, in vehicle control, alcohol-induced change in artery diameter from pre-alcohol level averaged −1.03% ± 0.42% (Figures 3A,B). In presence of DHA, however, alcohol-induced change in artery diameter averaged 0.46% ± 0.20% (p = 0.03 by two-tailed Mann-Whitney test when compared to vehicle control, Figure 3B). In slo1Y450F knock-in males, alcohol-induced change in artery diameter averaged −0.61% ± 0.28% (Figures 3A,B). In presence of DHA, alcohol-induced change in artery diameter averaged −0.91% ± 0.10% (p = 0.31 by two-tailed Mann-Whitney test when compared to vehicle control; Figure 3B). Further analysis of data from male arteries using two-way ANOVA revealed significant main effect of DHA treatment (p = 0.04) and trending effect of genotype (p = 0.09). Most important, a significant interaction between genotype and DHA treatment was detected (p = 0.004), underscoring that the loss of antagonism on alcohol-induced cerebral artery constriction by DHA treatment is actually determined by the substitution of slo1Y450.

In females, alcohol-induced constriction was not readily observed. In vehicle control, alcohol-induced changes in diameter averaged 0.29% ± 0.59% (Figures 3C,D). In presence of DHA, fold-change in artery diameter by alcohol averaged 0.61% ± 0.35% (p = 0.94 by two-tailed Mann-Whitney test when compared to vehicle control, Figure 3D). Remarkably, middle cerebral arteries from slo1Y450F knock-in females exhibited a pronounced constriction by alcohol: averaged change in diameter by alcohol in this group reached −3.87% ± 0.69% (Figures 3C,D). DHA treatment significantly attenuated this response, reducing the change in artery diameter to 0.27% ± 0.24% (p = 0.002 by two-tailed Mann-Whitney test when compared to vehicle control, Figure 3D). Consistent with pair-wise comparisons, further analysis of data from female arteries using two-way ANOVA revealed significant main effects of genotype (p = 0.001) and DHA treatment (p = 0.002). A significant interaction between genotype and DHA treatment was also detected (p = 0.0008).

Collectively, our findings indicate that Y450 within the BK channel slo1 subunit is a sexually dimorphic sensor of DHA protection against alcohol. Furthermore, combined with previous reports on the critical role of slo1Y450 in mediating cholesterol protection against alcohol in cerebral arteries, slo1Y450 emerges as a potential relay station for cholesterol, DHA, and alcohol actions on cerebral artery diameter.

## Discussion

In the present study we aimed at addressing whether using two protective agents - cholesterol and DHA – offered a synergistic benefit against alcohol-induced constriction of cerebral arteries. The search for agents that counteract alcohol’s deleterious effects on cerebral circulation constitutes a relevant task, as blood alcohol levels reached during episodic moderate-to-heavy alcohol consumption constrict cerebral arteries in many species, including *Homo sapiens* [3]. The degree of alcohol-induced constriction varies with species [20] and sex [14]. In studies with rats, alcohol-induced constriction progressively increased as animals were reaching adulthood [21]. Disregarding some variation in the degree of alcohol-induced constriction of the cerebral circulation, this alcohol action would inevitably dampen local cerebral blood flow. Indeed, even small alterations in cerebral artery diameter will have a large impact on cerebral perfusion, as by Poiseuille’s law changes in flow are proportional to 4th power of the radius [22]. Thus, a 5% change in cerebral artery diameter will render up to 20% decrease in blood flow [20]. Halted blood supply will inevitably impact neuronal function, as neuronal activity is particularly sensitive to perturbation in cerebral vasculature [23–25].

During the study, we probed a range of alcohol concentrations between 5 and 100 mM, which are relevant from pharmacological and toxicological standpoints. Low alcohol doses, such as 5 mM exert mild effects on behavior, yet already perturb neuronal activity [26, 27]. 17 mM alcohol corresponds to blood alcohol level representing legal limit of intoxication to drive motor vehicle in most of the United States. 35–50 mM alcohol is reached in the blood during moderate-to-heavy drinking episodes [28]. Lastly, 75–100 mM represent sub-lethal blood alcohol levels in humans, with alcohol exceeding 100 mM being lethal in many cases [29]. At the range of alcohol concentrations studied (5–100 mM), middle cerebral arteries and their branches from male Sprague-Dawley rats exhibit concentration-dependent constriction *ex vivo* with maximal reduction in diameter reaching 6.4% ± 1.6% from pre-alcohol value at 100 mM alcohol [20]. Since 100 mM represents a sub-lethal dose in humans [29], our *in vivo* experiments utilized a lower dose of alcohol (50 mM; Figure 1), which is still expected to render measurable constriction of cerebral arteries *in vivo* and *ex vivo*. Infusion of this alcohol concentration into cerebral circulation of male Sprague-Dawley rat with naïve cholesterol renders on average 20% reduction in diameter of pial arteriole stemming from middle cerebral arteries [14]. Yet either high-cholesterol diet or daily DHA oral supplements ablate this *in vivo* effect of alcohol [10, 13].

In contrast to males, middle cerebral arteries and arterioles of female Sprague-Dawley rats are consistently less sensitive to alcohol [13, 14]. The reasons for such sex specificity of alcohol effect on cerebral artery diameter remain unstudied. One of the plausible explanations focuses on molecular target of alcohol in cerebral vasculature–BK channel [4]. Alcohol-induced constriction of cerebral arteries is enabled by alcohol inhibition of cerebral artery myocyte BK channels [4]. Moreover, at physiological levels of Ca^2+^_i_ in the contracting myocyte, that is, when BK channel activity operates as a negative feed-back on contraction [30], alcohol inhibits BK channels only when multimeric channel complexes contain accessory β1 subunit [31]. Thus, lack of alcohol sensitivity in arteries from females may potentially result from a lack of β1 subunit within cerebrovascular smooth muscle BK channel complexes or, in contrast, their over-abundance. The latter was documented to limit alcohol-induced constriction in rat cerebral arteries following loading of arteries with BK β1 subunit-coding cDNA [20]. Last but not least, female cerebral arteries may simply have a deficient expression of BK channels. Modulation of BK channel activity by steroids, including female steroids estrogen and progesterone is widely reported [32–35].

Our probing of 50 mM alcohol on cerebral arteriole diameter *in vivo* following the high-cholesterol diet did not render vasoconstriction, and this lack of sensitivity to alcohol was observed in both, males and females (Figure 1). This is consistent with previous findings that showed a protective role of cholesterol accumulation within artery wall against alcohol effect on cerebral artery diameter [10, 36]. Molecular mechanisms that enable cholesterol-driven protection against alcohol effect on cerebral artery diameter have been a focus of investigation by our group for several years. For cholesterol, we first established that protection against alcohol inhibition of BK channels and cerebral vasoconstriction did not require BK channel accessory β1 subunit [37]. Furthermore, we established that protection was enabled by Y450 within cerebrovascular myocyte BK channel-forming subunit (slo1) [12]. Tyrosine substitution for phenylalanine disabled the protective effect of cholesterol [12]. Remarkably, Y450 was earlier postulated to participate in cholesterol docking on BK channel [38]. However, it is unlikely that cholesterol protection against alcohol is solely enabled by cholesterol docking to Y450. Two pieces of evidence argue against such possibility. First, enrichment with enantiomeric cholesterol still provides protection against alcohol [37], yet enantiomeric cholesterol is unable to support cholesterol-characteristic (Y450-mediated) inhibition of slo1 currents [19]. Second, cholesterol protection against alcohol is only observed when cholesterol is delivered into myocyte with preserved intracellular content, not isolated membrane patch, and is mediated by a protein kinase C-related mechanism [11]. Therefore, cholesterol protection against alcohol involves a cellular pathway that is sensed by Y450 within BK channel-forming subunit.

Regarding DHA, we were able to determine that DHA protection against alcohol effect on cerebral artery diameter could not be observed in slo1 subunit-lacking mouse [13]. However, we could not establish specific amino acid that enables DHA protection against alcohol. Mutation of a previously identified DHA-sensing amino acids on BK channel slo1 subunit (Y323 on rat cerebrovascular BK channel isoform that is equivalent to hslo1 Y318 [39]) did not ablate protection against alcohol-induced constriction [13]. In the present work, we hypothesized that lack of additive protection against alcohol when cholesterol and DHA are co-administered (Figures 2B,E) resulted from competitive interaction of these two lipids on a common molecular target. Consistent with the critical role of slo1Y450 in cholesterol-sensing and its protective action against alcohol [12, 38], slo1Y450 could be a common molecular target that also enables DHA-driven protection against alcohol. This hypothesis receives support by our findings in middle cerebral arteries from male mice (Figure 3B). Specifically, slo1Y450F knock-in led to lost protection by DHA when arteries from these mice were probed with 50 mM alcohol (Figure 3B). This outcome supports the idea that cholesterol and DHA may share a molecular pathway that enables protection against alcohol-induced cerebrovascular constriction. This pathway includes slo1 Tyr450 as a potential relay station for cholesterol, DHA and alcohol cerebrovascular effect which appears to be operant only in male animals.

Indeed, a particularly intriguing finding of the present work, however, is the sex-specific effect of the slo1Y450F knock-in on alcohol-induced cerebrovascular responses and resulting statistical outcome of DHA treatment. In slo1Y450F knock-in females, alcohol-induced constriction was exacerbated under vehicle control condition when compared to males (Figure 3D). Moreover, DHA effectively protected against alcohol constriction of cerebral arteries from female slo1Y450F knock-in mice. These sexually dimorphic effects suggest that slo1Y450 participates in sex-specific regulatory mechanisms that govern alcohol sensitivity of cerebrovascular BK channels. Given the established influence of sex hormones on membrane lipid composition, BK channel auxiliary subunit expression, and channel gating [15, 34, 40–44], it is plausible that loss of Y450 differentially alters the integration of alcohol-sensitive signaling pathways in males versus females. Future studies will be required to examine the molecular basis of observed sex-specific effects.

In conclusion, the present work demonstrates that combining cholesterol enrichment with DHA supplementation does not provide additional protection against alcohol effect on cerebral artery diameter, either *in vivo* or *ex vivo*. Lack of additivity or synergism in the protective actions of cholesterol and DHA against alcohol-induced cerebral artery constriction points at the possibility of functional convergence between these two lipid regulators of alcohol action. While cholesterol-dependent protection requires the BK channel slo1 subunit residue Y450, the role of this amino acid in mediating DHA protection against alcohol is sexually dimorphic. Together, our findings advance understanding of lipid–ion channel interactions in alcohol-induced cerebrovascular dysfunction and highlight the importance of considering sex-specific mechanisms when developing strategies to mitigate the vascular consequences of alcohol exposure.

## Data Availability

The raw data supporting the conclusions of this article will be made available by the authors, without undue reservation.
